# The Mechanistic Role of PPAR*γ* in Wound Healing

**DOI:** 10.1155/ppar/2242856

**Published:** 2026-01-28

**Authors:** Zhaojun Wang, Kaixing Jia, Wei Wang, Xiaolong Du, Yueyan Wu, Jianan Wang

**Affiliations:** ^1^ Department of Trauma Repair Reconstructive Surgery, Fenyang Hospital of Shanxi Province, Fenyang Hospital Affiliated to Shanxi Medical University, Fenyang College of Shanxi Medical University, Fenyang City, Shanxi Province, China, sxmu.edu.cn; ^2^ Fenyang College of Shanxi Medical University, Shanxi University of Medicine, Lvliang City, Shanxi Province, China, sxmu.edu.cn

**Keywords:** adipose tissue regeneration, macrophage polarization, PPAR*γ*, scar formation, wound healing

## Abstract

The receptor known as peroxisome proliferator‐activated receptor gamma (PPAR*γ*) is crucial for effective wound healing, and recent progress has given a deeper understanding of its complex functions. As a biological switch, PPAR*γ* regulates the immune response by shifting macrophages from promoting inflammation to supporting tissue regeneration, while suppressing pro‐inflammatory signals to create an ideal healing environment. At the cellular level, PPAR*γ* enhances the migration of keratinocytes and promotes re‐epithelialization, thereby accelerating the wound closure process. It also promotes the differentiation of preadipocytes and the formation of new blood vessels, making a significant contribution to tissue regeneration. At the molecular level, PPAR*γ* plays a dual role in guiding epithelial–mesenchymal transformation to aid healing while preventing excessive scarring. It improves mitochondrial efficiency to provide the energy needed for tissue repair. Despite these promising mechanisms, the clinical use of current PPAR*γ* agonists faces hurdles due to side effects and regulatory hurdles. Moving forward, research should aim to develop targeted delivery methods, tailor therapies to individual needs, and investigate how PPAR*γ* interacts synergistically with other signaling pathways, all of which are essential steps toward translating these findings into clinical practice.

## 1. Introduction

Wound healing represents a sophisticated biological cascade that coordinates multiple cellular and molecular events to restore tissue integrity following injury. This meticulously orchestrated process—spanning inflammatory control, cellular regeneration, blood vessel formation, and extracellular matrix (ECM) reorganization—determines the functional recovery of damaged tissue. While superficial wounds generally follow an efficient, self‐regulated healing trajectory, more severe injuries caused by major trauma, systemic diseases like diabetes, or persistent infections often face complications such as impaired healing and excessive scarring. These issues not only compromise the skin’s protective function but may also result in cosmetic concerns and physical limitations, dramatically affecting patients’ wellbeing [1, 2]. Consequently, unraveling the molecular drivers of wound repair and discovering therapeutic avenues to optimize this process remain key priorities in both trauma care and regenerative medicine.

PPAR*γ*, a key player in the nuclear receptor family, was first identified for its central role in fat cell development and metabolic balance. However, subsequent research has revealed its far‐reaching influence across diverse biological functions, from immune modulation and cell specialization to tissue regeneration. Emerging evidence highlights PPAR*γ*’s critical role in wound healing [3, 4]: it fine‐tunes the inflammatory response by steering macrophages toward a healing phenotype [5], accelerates wound closure by boosting keratinocyte movement and skin layer renewal [6], promotes tissue rebuilding by stimulating fat cell maturation [7] and blood vessel growth [8], and dynamically regulates epithelial–mesenchymal transition (EMT) [9] to balance repair and scarring [10]. These findings position PPAR*γ* as a multifaceted regulator of wound healing, offering promising theoretical support for novel therapeutic approaches.

The activity of PPAR*γ* is complex and context‐dependent, shaped by factors such as ligand binding, cell type, and environmental cues. It can trigger gene expression through direct DNA interaction after forming a heterodimer with RXR and binding to PPREs [11] or indirectly influence signaling pathways (e.g., NF‐*κ*B and STAT6) to modulate inflammation and metabolism [12]. Although traditional PPAR*γ* activators like thiazolidinediones (TZDs) [13, 14] have shown potential, their clinical use is hampered by adverse effects, including cardiovascular risks and metabolic disturbances. This underscores the importance of refining PPAR*γ*‐targeted therapies through deeper mechanistic insights.

This review synthesizes current knowledge on PPAR*γ*’s role in wound healing, detailing its regulatory effects on immune cells, skin cells, fat cells, and blood vessel linings. It also explores its interplay with inflammatory signals, metabolic pathways, and scar formation. By evaluating both the opportunities and challenges in clinical translation, this work aims to provide a robust scientific framework for developing PPAR*γ*‐based interventions in wound management.

## 2. The Wound Healing Process

Wound healing progresses through sequential, overlapping phases: inflammation, proliferation, and remodeling. Briefly, upon injury, hemostasis and inflammation initiate the process, involving platelet aggregation and neutrophil infiltration for debris clearance. The proliferative phase is characterized by fibroblast activation, ECM deposition, angiogenesis, and re‐epithelialization. Finally, the remodeling phase involves ECM maturation and scar formation. PPAR*γ* exerts regulatory functions across all these stages, as detailed in the following sections [15, 16].

## 3. Distribution and Physiological Significance of PPAR*γ* in Skin Organs

### 3.1. Overview of the Structure, Ligands, and Functions of PPAR*γ*

In the 1960s, researchers initially observed that the lipid‐lowering medication clofibrate could prompt an abnormal proliferation of peroxisomes, which are organelles involved in lipid oxidation, in the livers of rats. Various compounds, including fatty acids and fibrate lipid‐lowering drugs, can elicit similar effects and are collectively known as “peroxisome proliferators” (PP). In 1990, British scientists Issemann and Green isolated a novel nuclear receptor protein from mouse livers that could be selectively activated by PP [17]. This protein was termed peroxisome proliferator‐activated receptor (PPAR). Three subtypes of PPAR have been recognized: PPAR*α* (NR1C1), PPAR*δ*/*β* (NR1C2), and PPAR*γ* (NR1C3). As a member of the nuclear receptor superfamily, PPAR is a transcription factor that modulates gene expression upon ligand activation, regulating genes responsible for metabolic balance and diverse organ functions [18, 19]. PPAR*γ* is predominantly expressed in the immune system, adipose tissue, duodenum, and proximal colon, and is the most extensively studied subtype within the PPAR family. The human PPAR*γ* gene is situated in the 3p24.2‐p25 region of chromosome 3 and comprises nine exons [20]. Because of variances in promoters and alternative splicing, two isoforms of PPAR*γ* exist: PPAR*γ*1, expressed widely in various tissues (including white and brown adipose tissues, the immune system, liver, and muscle) and PPAR*γ*2, exclusive to adipose tissue, featuring an additional 28–30 amino acids at its N‐terminus compared to PPAR*γ*1 [21]. The structure of the PPAR*γ* protein is evolutionarily conserved, with the DNA‐binding ability concentrated in the highly conserved C domain [22].

PPARs modulate gene transcription via ligand‐induced conformational changes, forming heterodimers with retinoic X receptors to bind PPAR response elements [23]. Upon ligand binding, coactivators are recruited to initiate target gene expression. For instance, PPAR*γ* activation by fatty acid derivatives enhances lipid breakdown, while TZD drugs induce adipocyte differentiation [11, 24, 25]. Conversely, antagonists recruit corepressors to impede transcription. Some ligands can also elicit effects via PPAR‐independent pathways; for example, 15‐deoxy‐*Δ*12,14‐prostaglandin D2 inhibits nuclear factor *κ*B activity [26]. Phosphorylation, in addition to binding to response elements, modulates PPAR activity. Various kinases (e.g., MAPK, PKC, PKA) influence PPAR function through phosphorylation, with outcomes contingent upon multiple factors such as stimulus type, kinase involved, PPAR subtype, modified residue, cell type, and target gene promoter. The interplay of ligand binding and kinase‐mediated phosphorylation finely tunes PPAR activity [27, 28], integrating lipid and cell membrane signals to precisely regulate target genes in diverse physiological contexts through subtype‐specific modifications.

### 3.2. Distribution and Function of PPAR*γ* in Skin Tissue

In humans, PPAR*γ* demonstrates pronounced expression predominance in various tissues, notably white adipose tissue, colon, spleen, lymphoid tissue, and bone marrow [29, 30]. Additionally, notable expression is observed in the kidneys (particularly in the medullary collecting ducts and papillary urothelial cells), heart, small intestine, ovaries, testes, liver, bladder (transitional epithelial cells), and epidermal keratinocytes [6, 31, 32]. PPAR*γ*1 exhibits widespread expression in human tissues, particularly in macrophage lines and monocytes, where it plays a pivotal role in regulating genes associated with lipid metabolism and inflammatory responses [33]. Moreover, it is present in aortic and coronary artery smooth muscle cells, endothelial cells, and skeletal muscle [34]. The relative expression levels of PPAR*γ*1 and *γ*2 in adipose tissue vary among individuals and can be modulated by dietary factors. While adipocytes display similar responses in insulin sensitivity and gene expression profiles upon activation of either PPAR*γ*1 or *γ*2, PPAR*γ*2 demonstrates greater potency in inducing adipogenesis at lower ligand concentrations [35]. Furthermore, the distinct tissue distribution of the two isoforms, coupled with variations in their ratio, implies that their expression levels may be modulated by specific disease conditions or reflect the activation or deactivation of PPAR*γ* during disease progression [36].

The epidermis, the outermost layer of the skin, is a multilayered epithelium crucial for protecting against microbial, mechanical, and chemical threats. Keratinocytes, originating in the basal layer, undergo a sophisticated differentiation process as they migrate towards the stratum corneum. This process involves biochemical modifications, expression of structural proteins, and lipid processing reorganization, culminating in the formation of the hydrophobic stratum corneum. PPAR*γ*, present in various skin cell types, exhibits widespread expression in the skin [37, 38]. Its expression peaks during late embryonic development but declines postnatally [30, 32, 39, 40]. In adult skin, PPAR*α* and PPAR*γ* exhibit relatively low expression levels, while PPAR*β* is predominant. Despite lower expression levels in the dermis compared with the epidermis, PPAR*α*, *β*, and *γ* are also present in the dermis [19, 41]. PPAR*γ* plays a critical role in regulating the skin barrier by inhibiting the expression of pro‐inflammatory genes through antagonizing inflammatory transcription factors NF‐*κ*B and AP‐1 [40, 42].

PPAR*γ* is predominantly localized in keratinocytes within the skin [6, 41], with its expression increasing as keratinocytes transition from the basal to the granular layer [28]. In vitro studies have demonstrated that PPAR*γ* agonists can upregulate genes associated with keratinocyte differentiation [6, 42]. Immunohistochemical analysis reveals nuclear localization of PPAR*γ*, with variations in staining intensity observed among different cell types [28]. Positive staining for PPAR*γ* is also evident in the epidermis, the cytoplasm of the inner root sheath of hair follicles, sebaceous glands [43, 44], human melanocytes, and adipocytes within subcutaneous adipose tissue.

PPAR*γ* plays a pivotal role in preserving the structural and functional integrity of the skin by orchestrating multiple key processes [45, 46]. It facilitates the differentiation of epidermal keratinocytes, leading to the synthesis of essential structural proteins (e.g., loricrin, filaggrin) and barrier lipids (e.g., ceramides, cholesterol). This culminates in the formation of a robust cornified envelope and the secretion of lamellar bodies, crucial for establishing and maintaining the skin’s vital physical barrier, which effectively shields against water loss and external insults. Additionally, PPAR*γ* drives sebocyte differentiation and sebum production, essential for sebaceous gland function in skin moisturization and surface antibacterial defense, while also playing a role in regulating the hair follicle cycle.

In terms of immune modulation, PPAR*γ* exerts potent anti‐inflammatory effects by suppressing the production of pro‐inflammatory factors by keratinocytes and immune cells, as well as modulating immune cell functions to prevent exaggerated inflammatory responses. Furthermore, PPAR*γ* can enhance the skin’s resilience to oxidative stress induced by environmental factors like ultraviolet radiation by upregulating the expression of antioxidant enzymes. As a central regulatory node, PPAR*γ* is indispensable for maintaining overall skin homeostasis by comprehensively regulating barrier function, appendage activities, immune equilibrium, regenerative capacity, and antioxidant defenses. Dysregulation of PPAR*γ* is closely linked to the pathogenesis of various skin disorders.

Furthermore, the activation of PPAR*γ* plays a crucial role in orchestrating the wound‐healing process following skin injury. It enhances re‐epithelialization through promoting keratinocyte migration and proliferation, regulates ECM synthesis by fibroblasts, exerts moderate control over inflammation, and stimulates angiogenesis.

## 4. Mechanisms by Which PPAR*γ* Promotes Wound Healing

### 4.1. PPAR*γ* Affects Wound Healing by Regulating Immunity

#### 4.1.1. Anti‐Inflammatory Mechanism of PPAR*γ* During the Wound Healing Process

**Figure 1:**
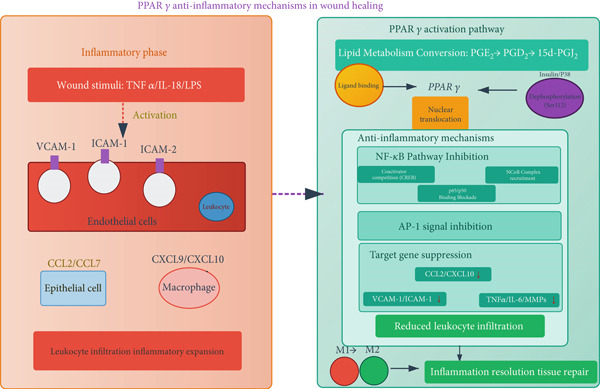
Anti‐inflammatory mechanisms of PPAR*γ* in wound healing. During the inflammatory phase, wound stimuli (TNF*α*/IL‐18/LPS) activate endothelial adhesion molecules (VCAM‐1/ICAMs), facilitating leukocyte infiltration and inflammatory amplification. In the repair phase, lipid metabolism shifts from PGE*₂* to PPAR*γ* ligands (PGD*₂*/15d‐PGJ*₂*). Insulin/P38‐mediated dephosphorylation (Ser112) activates PPAR*γ*, which suppresses inflammation through: (1) CREB coactivator competition; (2) p65/p50 DNA‐binding blockade; (3) NCoR complex recruitment to inhibit NF‐*κ* B; and (4) AP‐1 signal inhibition. This downregulates adhesion molecules, chemokines (CCL2/CXCL10), and inflammatory mediators (TNF*α*/IL‐6/MMPs), ultimately reducing leukocyte infiltration, promoting M1 *→* M2 macrophage transition, and resolving inflammation for tissue repair.

PPAR*γ* expression is dynamically regulated during wound repair (Figure 1). Its levels decrease initially post‐injury but significantly increase during the late repair stage, coinciding with a shift in lipid mediators from pro‐inflammatory prostaglandin E2 (PGE2) to anti‐inflammatory prostaglandin D2 (PGD2) and its metabolite 15‐deoxy‐*Δ*12,14‐prostaglandin J2 (15d‐PGJ2), which serves as an endogenous PPAR*γ* ligand [47, 48]. This temporal association suggests a role for the PGD2/15d‐PGJ2‐PPAR*γ* axis in inflammation resolution.

PPAR*γ* activation suppresses the expression of endothelial adhesion molecules (e.g., VCAM‐1) and chemokines (e.g., CCL2, CXCL10), thereby inhibiting leukocyte recruitment to the wound site [49, 50]. This anti‐inflammatory effect is mediated through multiple mechanisms, including: Transrepression of NF‐*κ*B and AP‐1: PPAR*γ* can directly interact with these pro‐inflammatory transcription factors or prevent the dissociation of corepressor complexes (e.g., NCoR), thereby suppressing the expression of their target genes [50, 51]. Ligand‐dependent mechanisms: Natural (e.g., 15d‐PGJ2) and synthetic (e.g., TZDs) PPAR*γ* ligands exert potent anti‐inflammatory effects in wounds, reducing pro‐inflammatory cytokines (TNF‐*α*, IL‐1*β*, IL‐6) and elevating anti‐inflammatory IL‐10. These effects are often reversible by PPAR*γ* antagonists like GW9662, confirming receptor specificity [52, 53].

#### 4.1.2. Regulatory Effect of PPAR*γ* on Immune Cells During Wound Healing

PPAR*γ* modulates the function of various immune cells to fine‐tune the wound immune landscape.

Dendritic cells (DCs): PPAR*γ* activation impairs DC maturation, reduces the expression of co‐stimulatory molecules (CD80, CD83) and IL‐12 secretion, thereby attenuating their antigen‐presenting capacity and subsequent T cell activation [54, 55].

T cells: PPAR*γ* activation in T cells suppresses the production of pro‐inflammatory cytokines like IFN*γ* (Th1) and IL‐17 (Th17), while promoting the differentiation and function of regulatory T cells (Tregs) [56, 57].

Mast cells and ILC2s: PPAR*γ* suppresses IgE‐mediated mast cell degranulation and inflammatory cytokine release [58]. It also inhibits the IL‐33‐driven production of IL‐5 and IL‐13 by group 2 innate lymphoid cells (ILC2s), curbing Th2‐type inflammation [59].

Monocytes/macrophages: (Discussed in detail in Section 4.1.3).

#### 4.1.3. Effects of PPAR*γ* on Macrophages

**Figure 2:**
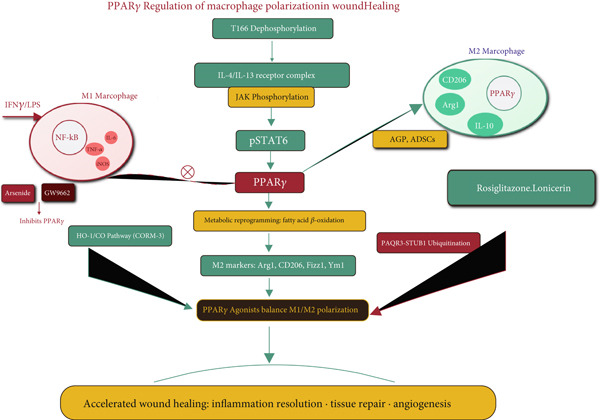
The role of PPAR*γ* in macrophage dynamics during wound healing. This schematic illustrates the central role of PPAR*γ* in macrophage polarization during wound healing. Pro‐inflammatory M1 macrophages secrete TNF‐*α*, IL‐6, and iNOS under IFN*γ*/LPS stimulation. PPAR*γ* activation via the IL‐4/IL‐13/pSTAT6 axis drives metabolic reprogramming (fatty acid *β*‐oxidation) and M2 polarization, enhanced by HO‐1/CO, T166 dephosphorylation, and PAQR3‐STUB1‐mediated stabilization. M2 macrophages express Arg1/CD206 and secrete IL‐10/TGF‐*β*1, promoting tissue repair and angiogenesis. PPAR*γ* agonists (rosiglitazone, lonicerin) balance M1/M2 polarization while inhibiting NF‐*κ*B, accelerating inflammation resolution and wound healing. Inhibitors (arsenide, GW9662) block this transition.

Macrophages are pivotal in wound healing, undergoing a phenotypic switch from pro‐inflammatory M1 to pro‐repair M2 states (Figure 2). PPAR*γ* is a master regulator of this M2 polarization [52, 60]. Drivers of M2 polarization: The IL‐4/IL‐13/STAT6 signaling pathway is a key inducer of PPAR*γ* expression in macrophages. PPAR*γ*, in turn, sustains the M2 phenotype and drives the expression of characteristic M2 markers like Arg1 and CD206 [61, 62]. Metabolic reprogramming: PPAR*γ* activation promotes fatty acid *β*‐oxidation and mitochondrial biogenesis, metabolic changes essential for alternative activation, and the repair functions of M2 macrophages [63].

Regulatory networks: Several pathways converge on PPAR*γ* to regulate M2 polarization. The HO‐1/carbon monoxide (CO) axis upregulates PPAR*γ* to promote M2 polarization [64]. The E2F1 transcription factor acts as a negative regulator of PPAR*γ*; E2F1 deficiency enhances PPAR*γ* expression and accelerates wound healing via M2 macrophages [65]. The PAQR3‐STUB1 axis controls PPAR*γ* protein stability via ubiquitination; knocking down PAQR3 stabilizes PPAR*γ*, enhances M2 polarization, and improves diabetic wound healing [66]. PPAR*γ* T166 dephosphorylation in macrophages triggers a lipid synthesis program that supports the secretion of reparative factors, a process critical for tissue repair [67].

### 4.2. Effects of PPAR*γ* on Keratinocytes

**Figure 3:**
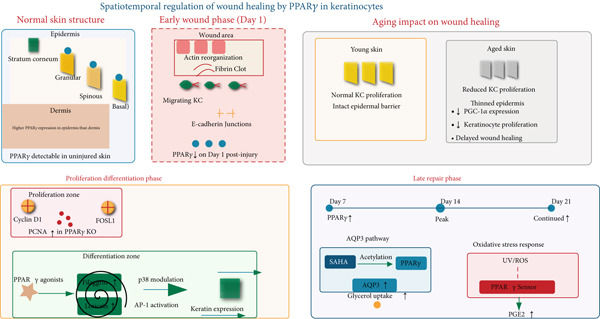
Spatiotemporal regulation of wound healing by PPAR*γ* in keratinocytes. PPAR*γ* orchestrates keratinocyte functions during wound repair: (1) Decreases on Day 1 post‐injury; (2) promotes migration via cytoskeletal reorganization; (3) enhances proliferation (Cyclin D1/FOSL1) but PCNA increases in PPAR*γ* KO; (4) induces differentiation markers (filaggrin/loricrin) via AP‐1/p38; (5) upregulates AQP3 and glycerol uptake; (6) mediates oxidative stress responses. Aging reduces PGC‐1*α*, impairing proliferation and delaying healing. Arrows indicate activation pathways.

PPAR*γ* is expressed in keratinocytes and its activation is crucial for multiple aspects of re‐epithelialization (Figure 3).

Promotion of differentiation: PPAR*γ* agonists upregulate the expression of differentiation markers (e.g., filaggrin, loricrin) in human keratinocytes, reinforcing the epidermal barrier [6, 42].

Enhancement of hydration: PPAR*γ* activators stimulate aquaporin‐3 (AQP3) expression and glycerol uptake in keratinocytes, contributing to skin hydration [68].

Dynamic expression in healing: PPAR*γ* expression in keratinocytes decreases initially after wounding but rises significantly in the later stages of repair, suggesting a primary role in the proliferative and remodeling phases [47].

Metabolic support: The PPAR*γ*‐PGC‐1*α* axis maintains NAD+ homeostasis and mitochondrial function in keratinocytes, which is critical for their proliferation and migration during aging and wound repair [69].

### 4.3. PPAR*γ* Regulates Adipocyte Differentiation to Accelerate Wound Healing

**Figure 4:**
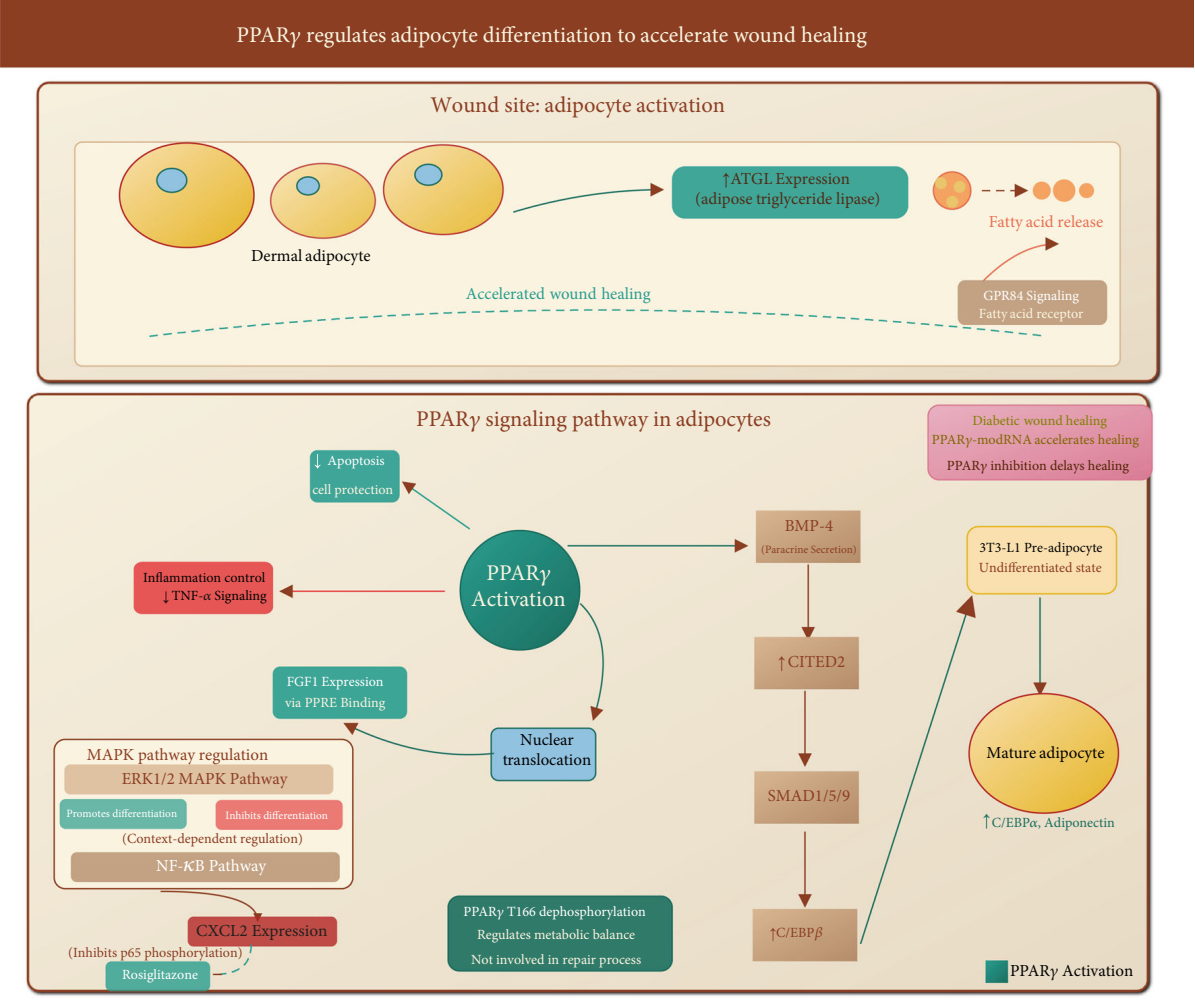
PPAR*γ*‐mediated adipocyte differentiation accelerates wound healing through multi‐pathway regulation. PPAR*γ* activation promotes nuclear translocation to induce FGF1 expression via PPRE binding, drives pre‐adipocyte differentiation with upregulated C/EBP*α*/adiponectin, and suppresses TNF‐*α* inflammation/apoptosis. BMP‐4 enhances adipogenesis through CITED2‐SMAD‐C/EBP*β* signaling. MAPK pathways exhibit context‐dependent regulation of differentiation, while NF‐*κ*B modulates CXCL2 expression inhibited by rosiglitazone. PPAR*γ*‐modRNA specifically accelerates diabetic wound healing. Fatty acid release via ATGL activates GPR84 signaling in wound microenvironment. T166 dephosphorylation regulates metabolic balance independently.

Dermal adipocytes play an active role in wound repair, and PPAR*γ* is the master regulator of adipocyte differentiation (Figure 4).

Induction of adipogenesis: PPAR*γ* activation drives the differentiation of preadipocytes into mature adipocytes, upregulating genes like C/EBP*α* and adiponectin [7, 25]. Chemically modified PPAR*γ* mRNA (PPAR*γ*‐modRNA) has been shown to accelerate wound healing by enhancing adipogenesis [7].

Modulation of the wound microenvironment: Upon skin injury, adipocytes increase lipolysis via adipose triglyceride lipase (ATGL), releasing fatty acids that can activate macrophages and other cells via G‐protein‐coupled receptors (e.g., GPR84), thereby influencing the wound milieu [70, 71].

Cross‐talk with other pathways: Adipogenic signals like BMP‐4 can enhance PPAR*γ*‐mediated differentiation through SMAD signaling and the coactivator CITED2 [10].

### 4.4. The Role of PPAR*γ*‐Regulated Angiogenesis in Wound Healing

PPAR*γ* exerts context‐dependent effects on angiogenesis, generally promoting physiological repair while potentially inhibiting pathological vessel growth.

Pro‐angiogenic effects: PPAR*γ* activation can enhance angiogenesis by upregulating VEGF expression in vascular smooth muscle cells and macrophages [72], and by improving the function of endothelial progenitor cells (EPCs) [73]. In diabetic models, PPAR*γ* agonists can partially restore impaired angiogenesis [74].

Dual regulation and specificity: The pro‐angiogenic effect of factors like FGF21 on brain microvascular endothelial cells requires PPAR*γ* activation [75]. Conversely, in specific contexts like the porcine placenta, PPAR*γ* can either stimulate or inhibit angiogenesis depending on the VEGF isoforms involved [76]. In corneal injury, PPAR*γ* activation can reduce pathological neovascularization [77].

### 4.5. PPAR*γ* Affects Wound Healing by Regulating Metabolism and Mitochondrial Function

Cellular metabolism and mitochondrial dynamics are reprogrammed during wound healing, and PPAR*γ* is a key modulator of this process.

Enhancement of mitochondrial function: PPAR*γ* agonists enhance mitochondrial biogenesis, oxidative phosphorylation efficiency, and antioxidant capacity, often through upregulating PGC‐1*α* [63, 69, 78].

Metabolic support for repair: In keratinocytes, the PPAR*γ*‐PGC‐1*α* axis maintains NAD+ levels and promotes fatty acid oxidation, providing energy and biosynthetic precursors crucial for re‐epithelialization. Age‐related decline in this axis contributes to healing deficiencies [69].

Anti‐inflammatory metabolic shift: In macrophages, PPAR*γ*‐driven fatty acid oxidation supports the anti‐inflammatory, pro‐repair M2 phenotype [63].

## 5. PPAR*γ* Affects Wound Healing by Regulating Epithelial–Mesenchymal Transition

Epithelial–mesenchymal transition (EMT) is a process where epithelial cells acquire mesenchymal traits, contributing to wound re‐epithelialization and, if dysregulated, to fibrosis. PPAR*γ* exerts a dual and context‐dependent role in regulating EMT.

Inhibition of profibrotic EMT: PPAR*γ* and its agonists can inhibit TGF‐*β*‐induced EMT in various models, thereby reducing fibrosis and scar formation. This is achieved by interfering with SMAD signaling and other profibrotic pathways [9, 79, 80].

Context‐dependent effects: While generally anti‐fibrotic, PPAR*γ* can sometimes promote EMT in certain tumor microenvironments through mechanisms involving TGF*β*1 upregulation or other pathways [81]. This highlights its nuanced role in tissue plasticity.

## 6. The Involvement of PPAR*γ* in Modulating Wound Healing and Inhibiting Scar Formation Through the Regulation of Fibroblasts

**Figure 5:**
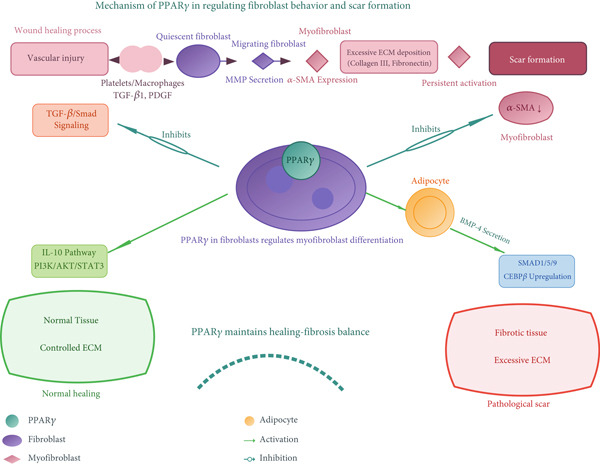
PPAR*γ* regulates scar formation via fibroblast modulation. Upon vascular injury, TGF‐*β*1/PDGF activates fibroblast migration and myofibroblast differentiation (*α*‐SMA↑), driving ECM deposition. Intracellular PPAR*γ* in fibroblasts: (1) Inhibits TGF‐*β*/Smad signaling by blocking p300; (2) activates IL‐10/PI3K/AKT/STAT3 pathway; (3) promotes adipocyte‐derived BMP‐4 secretion, inducing SMAD1/5/9/CEBP*β*. This triad suppresses myofibroblast activation and ECM overproduction (Collagen III/fibronectin↓), maintaining healing‐fibrosis balance to prevent pathological scarring.

During healing, fibroblasts differentiate into contractile myofibroblasts that drive wound contraction and ECM deposition. The persistence of myofibroblasts leads to excessive scarring (Figure 5). PPAR*γ* acts as a potent endogenous antifibrotic factor.

Suppression of myofibroblast activation: PPAR*γ* activation in skin fibroblasts counteracts the profibrotic effects of TGF‐*β*1, reducing the expression of *α*‐smooth muscle actin (*α*‐SMA) and ECM components like collagen. This occurs through mechanisms such as disrupting the TGF‐*β*/Smad pathway and enhancing IL‐10 production [82, 83].

Paracrine regulation by adipocytes: Adipocyte‐derived factors, notably BMP‐4, can activate PPAR*γ* in fibroblasts and promote myofibroblast dedifferentiation via the SMAD1/5/9 pathway and CITED2, offering a novel mechanism for scar inhibition [10].

Evidence in other tissues: PPAR*γ*’s anti‐scarring properties are also evident in corneal healing, where it reduces corneal haze and fibrosis by inhibiting fibroblast migration and ECM synthesis [84, 85].

## 7. Clinical Application Prospects and Challenges of PPAR*γ*

### 7.1. Promising Preclinical Strategies

Current research explores innovative approaches to harness PPAR*γ* for wound repair. These include the use of chemically modified PPAR*γ* mRNA (PPAR*γ*‐modRNA) to directly and transiently boost PPAR*γ* expression in adipocytes, enhancing tissue regeneration [7]. Another strategy focuses on developing biomaterials that leverage PPAR*γ*’s role in macrophage polarization to shift the wound microenvironment from pro‐inflammatory to pro‐regenerative [86].

### 7.2. Challenges in Clinical Translation

The translation of PPAR*γ*‐targeted therapies faces significant hurdles. The foremost is safety concerns associated with conventional TZD agonists (e.g., rosiglitazone, pioglitazone), which include weight gain, edema, cardiovascular risks, and bone loss, limiting their use for chronic conditions like wound healing [13, 87]. Furthermore, the mechanistic complexity of PPAR*γ*’s actions within the multicellular wound environment necessitates a deeper understanding for precise targeting.

### 7.3. Current Clinical Evidence and Trials

Contrary to the notion of a complete absence of clinical data, several clinical investigations have explored PPAR*γ* agonists in wound healing contexts. For instance, a study on pioglitazone in diabetic patients showed improved wound healing outcomes [88]. Preclinical studies further elucidate the mechanism, demonstrating that pioglitazone pretreatment enhances the therapeutic potential of stem cells in diabetic wound models. While these studies demonstrate proof‐of‐concept, they confirm the need for safer and more targeted PPAR*γ* modulators.

### 7.4. Future Directions

Future efforts should focus on the following: (1) Developing precise delivery systems (e.g., nanoparticles, topical formulations) to achieve local activation and reduce systemic exposure; (2) designing novel, safer agonists with improved selectivity; (3) personalizing therapies based on wound etiology and patient genetics; and (4) exploring combination therapies that target PPAR*γ* alongside other regenerative pathways.

## 8. Conclusion

**Figure 6:**
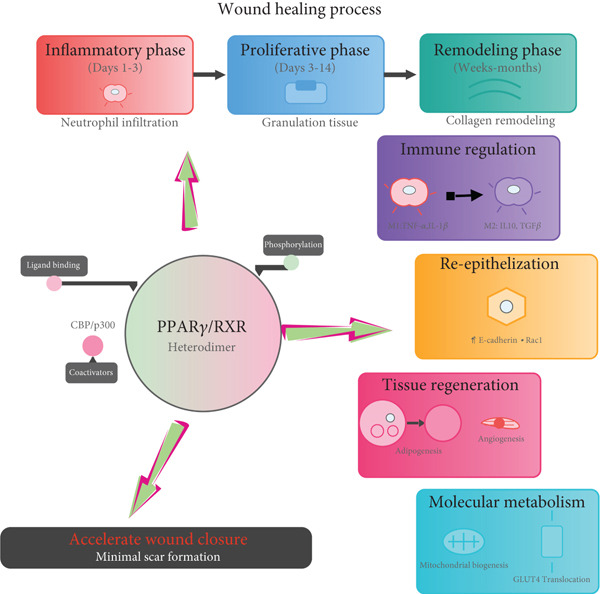
PPAR*γ* orchestrates wound healing. This schematic depicts PPAR*γ*’s central role across wound healing phases (Inflammatory, Proliferative, Remodeling). Upon ligand binding and RXR heterodimerization (with CBP/p300 coactivation), PPAR*γ*: (1) Polarizes macrophages from pro‐inflammatory (M1; TNF‐*α*, IL‐1*β*) to pro‐regenerative (M2; IL‐10, TGF‐*β*), suppressing inflammation; (2) accelerates re‐epithelialization via keratinocyte migration (↑E‐cadherin/Rac1) and wound closure; (3) drives tissue regeneration (angiogenesis, adipogenesis); (4) minimizes scarring while guiding repair and enhances metabolic support (mitochondrial biogenesis, GLUT4 translocation). Despite promoting granulation tissue and collagen remodeling, clinical translation of PPAR*γ* agonists faces challenges.

In conclusion, PPAR*γ* plays a crucial role in wound repair by orchestrating immune regulation, adipose tissue regeneration, and angiogenesis (Figure 6). Nevertheless, challenges such as the adverse effects of conventional agonists, unclear regulatory pathways in intricate microenvironments, and inadequate clinical evidence hinder its practical implementation. Future investigations should focus on creating sophisticated delivery systems through interdisciplinary partnerships and enhancing clinical verification alongside personalized medicine approaches. These efforts are essential for the successful transition of PPAR*γ*‐targeted therapy from experimental settings to clinical practice, ultimately improving outcomes for patients with wounds.

**Nomenclature**
AQP3: aquaporin 3
COX‐2: cyclooxygenase‐2
ECM: extracellular matrix
EMT: epithelial‐mesenchymal transition
EPCs: endothelial progenitor cells
HIF‐1*α*
: hypoxia‐inducible factor 1‐alpha
IL: interleukin
KCs: keratinocytes
MMPs: matrix metalloproteinases
OXPHOS: oxidative phosphorylation
PCNA: proliferating cell nuclear antigen
PDGF: platelet‐derived growth factor
PGC: peroxisome proliferator‐activated receptor *γ* co‐activator
PPAR*γ*
: peroxisome proliferator‐activated receptor gamma
PPRE: PPAR response element
TGF‐*β*
: transforming growth factor‐beta
TIMPs: tissue inhibitors of metalloproteinases
TNF‐*α*
: tumor necrosis factor‐alpha
TZDs: thiazolidinediones
VEGF: vascular endothelial growth factor

## Ethics Statement

The authors have nothing to report.

## Consent

All authors agree to publish this review.

## Disclosure

All authors have read and approved the final manuscript.

## Conflicts of Interest

The authors declare no conflicts of interest.

## Author Contributions

Jianan Wang, Zhaojun Wang, and Kaixing Jia contributed equally to this work and were mainly responsible for literature review and drafting the manuscript. Xiaolong Du, Yueyan Wu, Wei Wang, and Jianan Wang guided the research strategy, critically reviewed the intellectual content of the manuscript. All contributors are listed as authors; no unacknowledged individuals or third‐party services were involved.

## Funding

This study was supported by Lvliang City Key Research and Development Project (Social Development No. 2020SHFZ39); the Fenyang Hospital’s Scientific Research Program, No. 2024013 and No. 2024021.

## Data Availability

Data sharing not applicable to this article as no datasets were generated or analyzed during the current study.
